# Fatal Systemic Capillary Leak Syndrome after SARS-CoV-2Vaccination in Patient with Multiple Myeloma

**DOI:** 10.3201/eid2711.211723

**Published:** 2021-11

**Authors:** Gwang-Jun Choi, Seon Ha Baek, Junmo Kim, Jung Ho Kim, Geun-Yong Kwon, Dong Keun Kim, Yeon Haw Jung, Sejoong Kim

**Affiliations:** Daegu Metropolitan Government, Daegu, South Korea (G.-J. Choi);; Hallym University Dongtan Sacred Heart Hospital, Hwaseong, South Korea (S.H. Baek);; National Forensic Service Daegu Institute, Daegu (J. Kim);; Yeungnam University College of Medicine, Daegu (J.H. Kim);; Korea Disease Control and Prevention Agency, Cheongju, South Korea (G.-Y. Kwon, D.K. Kim, Y.H. Jung);; Seoul National University Bundang Hospital, Seongnam, South Korea (S. Kim)

**Keywords:** Ad26.COV2.S, Clarkson disease, coronavirus disease, COVID-19, COVID-19 vaccine, monoclonal gammopathy, multiple myeloma, respiratory infections, SARS-CoV-2, severe acute respiratory syndrome coronavirus 2, systemic capillary leak syndrome, vaccines, viruses

## Abstract

A young man with smoldering multiple myeloma died of hypotensive shock 2.5 days after severe acute respiratory syndrome coronavirus 2 vaccination. Clinical findings suggested systemic capillary leak syndrome (SCLS); the patient had experienced a previous suspected flare episode. History of SCLS may indicate higher risk for SCLS after receiving this vaccine.

Systemic capillary leak syndrome (SCLS) is an extremely rare disease of unknown incidence (*1*). Typical manifestations of SCLS include hypotension, edema, hemoconcentration, and hypoalbuminemia after nonspecific prodromal illnesses (*1*,*2*). Increased capillary vascular permeability is the commonly accepted pathophysiology (*1*,*2*). However, the exact pathogenesis remains unclear. 

As part of the efforts to combat the ongoing pandemic of coronavirus disease (COVID-19), caused by severe acute respiratory syndrome coronavirus 2, the US Food and Drug Administration on February 27, 2021, gave emergency use authorization to the Ad26.COV2.S vaccine (Johnson & Johnson/Janssen, https://www.jnj.com). An SCLS case series reported 1 case of SCLS in a patient who received the Ad26.COV2.S vaccine (*3*). The European Medicines Agency reviewed 3 cases of SCLS in Ad26.COV2.S vaccine recipients and issued a report, published July 9, 2021, advising against administering the vaccine in persons with previous SCLS experiences (*4*). We describe a case of SCLS after Ad26.COV2.S vaccination in a patient with smoldering multiple myeloma.

A 38-year-old man reporting vomiting and dizziness sought treatment at an emergency department. Smoldering multiple myeloma had been diagnosed 1.5 years before, but no laboratory abnormalities had been found in his most recent hospital visit 5 months earlier. He had received the Ad26.COV2.S vaccine 2 days before the emergency department visit and experienced fever, chills, and myalgia 12–24 hours postvaccination, then nausea, recurrent vomiting, and general weakness 24–48 hours postvaccination. At admission, he was afebrile, his heart rate was 130 beats/min, and his blood pressure was 100/90 mm Hg, with no noticeable edema. We administered isotonic saline and initiated diagnostic evaluations: laboratory tests, imaging, and COVID-19 reverse transcription PCR. Test results (Table) showed marked hemoconcentration and hypoalbuminemia. Chest and abdominal computed tomography results were unremarkable. Six hours after admission, the patient was hypotensive (blood pressure 60/40 mm Hg), had a heart rate of 132 beats/min, and reported dyspnea. We obtained blood cultures and treated the patient with broad-spectrum antimicrobials, intravenous fluids, and inotropes. Despite these measures, the patient’s hypotensive shock worsened, and he died 10 hours after admission.

**Table:** Results of laboratory tests in patient with smoldering multiple myeloma who had SCLS develop after vaccination for severe acute respiratory syndrome coronavirus, South Korea*

Clinical measures	Reference range	Test results after SCLS episodes	
		5 mo earlier	Postvaccination
Leukocytes, ×10^3^/mm^3^	4–10	6.88	29.42
Hemoglobin, g/dL	13–17	14.7	22.7
Hematocrit, %	40–52	44.3	63.7
Platelet, ×10^3^/mm^3^	140–440	259	133
Albumin, g/dL	3.5–5.0	4.8	3.3
Blood urea nitrogen, mg/dL	8–23	13.6	33
Creatinine, mg/dL	0.7–1.2	0.94	2.0
Aspartate transaminase, IU/L	10–35	22	30
Alanine transferase, IU/L	0–40	14	4
Total bilirubin, mg/dL	0.1–1.2	0.5	1.46
Calcium, mg/dL	8.6–10.6	10.0	8.9
Erythrocyte sedimentation rate, mm/h	0–20	Not done	13
C-reactive protein, mg/dL	0–0.5	Not done	2.371
Procalcitonin, ng/ml	0–5	Not done	0.641
Troponin I, ng/mL	0–0.04	Not done	0.017
Creatine kinase myocardial band, ng/mL	0.6–6.3	Not done	3.5
N terminal-pro B-type natriuretic peptide, pg/mL	0–125	35.1	4,427
Lactic acid, mmol/L	0.5–1.6	Not done	5.4
Creatine phosphokinase, IU/L	1–171	Not done	276

*Vaccine was Ad26.COV2.S (Johnson & Johnson/Janssen, https://www.jnj.com). SCLS, systemic capillary leak syndrome.

Although at admission the patient showed neither peripheral edema nor severe hypoalbuminemia, we suspected SCLS for several reasons. First, we could not entirely rule out infection, but results of blood cultures and COVID-19 testing were negative. Second, autopsy results showed no evidence of acute infection or cardiovascular disease in the internal organs. We identified pulmonary edema, pleural effusion, and pericardial effusion. Although pulmonary edema is atypical in acute SCLS attacks (leak phase), prolonged cardiopulmonary resuscitation and fluid administration might have affected the autopsy findings. Histopathologic findings in both kidneys suggested autolysis or acute tubular necrosis, which helped exclude other possible etiologies of refractory hypotensive shock. Third, through medical chart review, we found that the patient in our study had been admitted 1.5 years earlier for fever, vomiting, myalgia, generalized edema, and hypotension (blood pressure 90/60 mm Hg). Laboratory results showed hemoconcentration (hematocrit 58.4%) and hypoalbuminemia (3.03 g/dL at nadir), but diagnosis was unclear, and the patient recovered spontaneously after fluid administration. We retrospectively assumed a flare episode of SCLS. Fourth, ≈80% of patients with SCLS have monoclonal gammopathy of undetermined significance (MGUS) (*2*,*5*), and there have also been other reports of SCLS in patients with multiple myeloma (*2*). The patient who had the previous reported case of SCLS after Ad26.COV2.S vaccination had MGUS (*3*), and the patient in our study had multiple myeloma. Recently, an additional report described a patient with MGUS who experienced severe SCLS 2 days after receiving the ChAdOx1 nCOV-19 vaccine (Oxford/AstraZeneca, https://www.astrazeneca.com); that patient also had an unrecognized previous episode of presumed SCLS (*6*).

 We believe a life-threatening flare developed after COVID-19 vaccination in the patient in our study who had a history suggestive of SCLS. Clinical findings were compatible with a previous report in which life-threatening disease occurred 1–2 days after vaccination (*3*,*6*); we could identify no SCLS triggers other than receiving the COVID-19 vaccine. Data from a review article indicated that 44% of 134 patients had identifiable SCLS triggers; 88% of those were infections, usually respiratory, and 11% involved intense physical exertion or extended travel (*7*). There was also a case report of possible SCLS related to the influenza vaccine; although not clearly meeting all the criteria for SCLS, a peritoneal dialysis patient experienced recurrent episodes of hypotension, peripheral edema, and hypoalbuminemia after 2 consecutive seasons of influenza vaccination (*8*). Immunologic response to vaccination has been proposed as a possible mechanism (*8*), but further studies are needed to verify factors predisposing patients to SCLS after COVID-19 immunization.

In South Korea, 1,129,796 people had received the Ad26.COV2.S vaccine as of August 2, 2021 (*9*); we have found no other reports of possible SCLS in vaccine recipients in South Korea. Our report describes the clinical course and characteristics of SCLS after COVID-19 vaccination. SCLS is often difficult to diagnose and may be misdiagnosed as other diseases, such as culture-negative sepsis. Therefore, clinicians should be aware of possible SCLS, especially in at-risk populations, and medical histories should be examined before vaccine is administered. 
